# Adjunctive repetitive transcranial magnetic stimulation for adolescents with first-episode major depressive disorder: a meta-analysis

**DOI:** 10.3389/fpsyt.2023.1200738

**Published:** 2023-08-01

**Authors:** Chen-Hui Sun, Jian-Xin Mai, Zhan-Ming Shi, Wei Zheng, Wen-Long Jiang, Ze-Zhi Li, Xing-Bing Huang, Xin-Hu Yang, Wei Zheng

**Affiliations:** ^1^Qingdao Mental Health Center, Qingdao, China; ^2^The Affiliated Brain Hospital of Guangzhou Medical University, Guangzhou, China; ^3^Chongqing Jiangbei Mental Health Center, Chongqing, China; ^4^Xiamen Xian Yue Hospital, Xiamen, China; ^5^The Third People’s Hospital of Daqing, Daqing, China

**Keywords:** repetitive transcranial magnetic stimulation, adolescent, depression, first episode, meta-analysis

## Abstract

**Objective:**

This meta-analysis of randomized clinical trials (RCTs) was conducted to explore the therapeutic effects, tolerability and safety of repetitive transcranial magnetic stimulation (rTMS) as an adjunct treatment in adolescents with first-episode major depressive disorder (FE-MDD).

**Methods:**

RCTs examining the efficacy, tolerability and safety of adjunctive rTMS for adolescents with FE-MDD were included. Data were extracted by three independent authors and synthesized using RevMan 5.3 software with a random effects model.

**Results:**

A total of six RCTs involving 562 adolescents with FE-MDD were included. Adjunctive rTMS was superior in improving depressive symptoms over the control group [standardized mean difference (SMD) = −1.50, 95% confidence interval (CI): −2.16, −0.84; *I*^2^ = 89%, *p* < 0.00001] in adolescents with FE-MDD. A sensitivity analysis and two subgroup analyses also confirmed the significant findings. Adolescents with FE-MDD treated with rTMS had significantly greater response [risk ratio (RR) = 1.35, 95% CI: 1.04, 1.76; *I*^2^ = 56%, *p* = 0.03] and remission (RR = 1.35, 95% CI: 1.03, 1.77; *I*^2^ = 0%, *p* = 0.03) over the control group. All-cause discontinuations were similar between the two groups (RR = 0.79, 95% CI: 0.32, 1.93; *I*^2^ = 0%, *p* = 0.60). No significant differences were found regarding adverse events, including headache, loss of appetite, dizziness and nausea (*p* = 0.14–0.82). Four out of six RCTs (66.7%), showed that adjunctive rTMS was more efficacious over the control group in improving neurocognitive function (all *p* < 0.05).

**Conclusion:**

Adjunctive rTMS appears to be a beneficial strategy in improving depressive symptoms and neurocognitive function in adolescents with FE-MDD. Higher quality RCTs with larger sample sizes and longer follow-up periods are warranted in the future.

## 1. Introduction

As a common mental disorder, major depressive disorder (MDD) affects approximately 5–15% of children and adolescents (1). Depression during adolescence is associated with a high risk of academic failure and behavioral problems (2), suicidal ideation and attempts (3), and adverse mental health consequences (i.e., anxiety disorder and substance use disorder) in the future (4–6). As a result, developments in treating adolescents suffering from MDD may have a positive influence on public health.

The usual treatment modalities for adolescents with MDD, mainly psychotherapy (e.g., cognitive behavioral therapy [CBT]), pharmacotherapy (e.g., selective serotonin reuptake inhibitors [SSRIs]) or both (7, 8), remain limited. Previous studies have found that at least 40% of adolescents with MDD showed unsatisfactory responses to those treatments (9, 10). For instance, psychotherapy may involve substantial time and financial costs, which lead to poor treatment compliance (11), while pharmacotherapy for adolescent patients may be associated with adverse events and even increased suicide risk (12). Therefore, there is an urgent need to explore more efficient and acceptable therapeutics for adolescents with MDD in clinical practice.

Repetitive transcranial magnetic stimulation (rTMS), as a noninvasive physical therapy, can modulate brain network functioning by producing a local magnetic field that acts on the local cerebral cortex and depression-related areas (13). rTMS has received the US Food and Drug Administration (FDA) approval to treat MDD among adults rather than adolescents (9). Accumulating randomized controlled trials (RCTs) have revealed the positive therapeutic effects of rTMS in adult patients suffering from treatment-refractory depression (TRD) (14, 15). Growing evidence has shown that rTMS can also improve drug efficacy in adult patients with first-episode major depressive disorder (FE-MDD) (16). For adolescents with MDD, several open-label studies (17, 18) have shown adjunctive rTMS to be a potentially effective treatment. However, the findings of RCTs (19–24) examining the therapeutic effects and safety of adjunctive rTMS in the treatment of adolescents with FE-MDD were inconsistent.

Therefore, the main aim of this meta-analysis was to investigate the therapeutic effects, tolerability and safety of adjunctive rTMS for adolescents with FE-MDD. We hypothesized that active rTMS plus antidepressants would be more efficacious than sham rTMS plus antidepressants or antidepressant monotherapy in improving depressive symptoms in FE-MDD patients among adolescents.

## 2. Methods

### 2.1. Search strategy

Based on the Preferred Reporting Items for Systematic Reviews and Meta-Analyses (PRISMA) guidelines (25), three authors (CHS, XHY and ZMS) independently retrieved RCTs examining the efficacy, tolerability and safety of adjunctive rTMS for adolescents with FE-MDD in international (Cochrane Library, PubMed, PsycINFO, and EMBASE) and Chinese (Wan Fang and Chinese Journal Net databases) databases from the establishment of the database to 9 November 2022. The detailed search strategy is presented in Appendix S1. Furthermore, the reference lists of meta-analyses and review articles (1, 9, 26) and the included RCTs (19–24) were searched manually and independently by the same three investigators to identify additional studies.

### 2.2. Inclusion and exclusion criteria

The inclusion criteria were conducted based on the following *PICOS* principle. *P*articipants: the study subjects must be adolescent patients (aged ≥12 years and ≤ 18 years) with a diagnosis of FE-MDD based on standardized diagnostic interviews. Following the methodology of a recent systematic review (27), adolescents were defined as those who were 12–18 years old. According to the recommendations of a previous meta-analysis (28), the sample was considered an FE-MDD group if the literature showed explicit characteristic descriptions (e.g., first-episode depression, first-episode depressive disorder, early depression) for the enrolled patients. *I*ntervention: active rTMS plus antidepressants. *C*omparison: antidepressants plus sham rTMS or antidepressant monotherapy. *O*utcomes: the primary outcome was the improvement of depressive symptoms at the post-rTMS time point measured with standardized instruments, such as the Hamilton Depression Rating Scale (HAMD) (29). The secondary outcomes were (1) study-defined response (i.e., at least 50% reduction in HAMD scores) and remission (i.e., at least 75% reduction in HAMD scores); (2) discontinuation due to any reason; (3) adverse events; and (4) neurocognitive function. ***S*** tudy design: only published RCTs targeting the efficacy and safety of adjunctive active rTMS versus sham rTMS or antidepressant monotherapy for adolescents with FE-MDD were included. Thus, studies examining the efficacy and safety of active rTMS alone versus antidepressants (30) or sham rTMS alone (31) were excluded. Furthermore, studies involving of other inventions such as any kind of psychotherapy were excluded. Case reports/series, non-RCTs and reviews were excluded.

### 2.3. Data extraction

We established a standardized Microsoft Excel table to extract essential information from selected studies. This process was independently conducted by the same authors (XHY, CHS and ZMS). If there were some inconsistencies, they were resolved by discussion within the team or the involvement of a senior investigator WZ (from Guangzhou). If relevant data were missing in the included literature, the first and/or corresponding authors were contacted by email or telephone for accurate information. If the eligible RCT consisted of a mixture of FE-MDD and multiepisode MDD, only data from the FE-MDD group were extracted.

### 2.4. Quality assessment

Three authors (XHY, CHS, and ZMS) independently assessed the quality of each RCT using the Cochrane risk of bias (32) and the Jadad scale (33). A Jadad scale score < 3 was rated as ‘low quality’, and a Jadad scale score ≥ 3 was rated as ‘high quality’. The overall evidence level of meta-analyzable outcomes was evaluated by the grading of recommendations assessment, development, and evaluation (GRADE) system (34, 35).

### 2.5. Statistical analyses

We used Revman software (version 5.3) to compute primary and secondary outcomes through a random effects model (36). For dichotomous data and continuous data, the risk ratio (RR) and standardized mean difference (SMD) and their 95% confidence intervals (CIs) were calculated. Heterogeneity among different studies was determined using Cochrane’s *Q* and *I*^2^ test, with Q < 0.1 or *I^2^* ≥ 50% suggesting significant heterogeneity (37). We conducted a subgroup analysis for the primary outcome: high-frequency (>1 Hz) rTMS (HF-rTMS) targeting the left dorsolateral prefrontal cortex (L-DLPFC) versus low-frequency (≤1 Hz) rTMS (LF-rTMS) targeting the right dorsolateral prefrontal cortex (R-DLPFC). For the primary outcome, a sensitivity analysis was performed to explore the source of heterogeneity by removing one study (23) with an outlying effect size of −2.53. Publication bias was assessed using funnel plots and Egger’s regression interanalyses (38). In all analyses, *p* < 0.05 was defined as a significant difference (two-sided).

## 3. Result

### 3.1. Literature search

**Figure 1:**
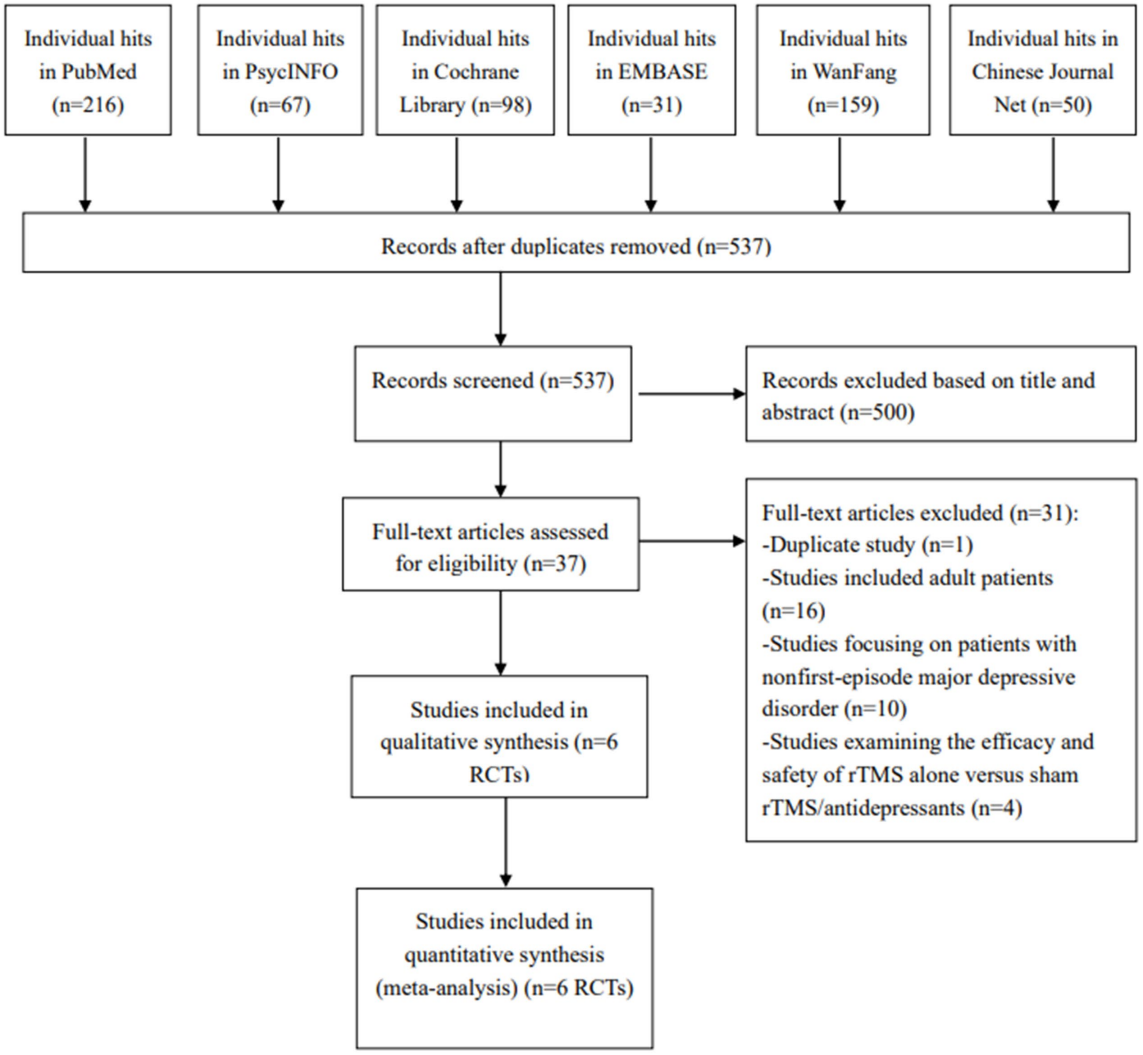
PRISMA flow diagram.

According to the search strategy, 621 studies were retrieved. After screening the title, abstract and full text, six RCTs (19–24) fulfilled the inclusion criteria and were analyzed in this meta-analysis (Figure 1).

### 3.2. Participant and study characteristics

As shown in Table 1, six RCTs were conducted in China covering 562 patients (281 patients in the rTMS group and 281 patients in the control group). The mean age was 15.0 years (range = 12–18 years). Male patients accounted for 36.5% (range = 18.6–51.3%) of the total sample. The use of antidepressants included sertraline (4 RCTs) (19, 21–23) and fluoxetine (1 RCT) (24). Participants underwent 1 Hz frequency rTMS (LF-rTMS) in 2 RCTs (20, 24) and 10 Hz frequency rTMS (HF-rTMS) in 4 RCTs (19, 21–23) (Table 1). The treatment duration of rTMS ranged from 2 to 6 weeks. The detailed treatment parameters of rTMS among the included RCTs are summarized in Table 1.

**Table 1:** Participant characteristics and rTMS parameters of each included study.

Study (country)	Number of Participants^a^	-Diagnostic criteria -Setting	Mean age (years)^b^ (range)	-Illness duration (months)^b^ -Male (%)	Treatment duration of rTMS (weeks)^c^	Intervention versus control groups: (dosage of antidepressants); the number of patients (n)	-Intensity (%MT) -Frequency (Hz)	Site	-Number of trains per day -Train duration (s) -Intertrain duration (s)	-Pulses per session -Number of sessions -Total pulses	Jadad score
Chen et al., 2022 (China)	100	-DSM-IV -In- and outpatients	15.0 (12–18)	-16.6 -18.6	2	1. Sertraline (50–100 mg/day) + rTMS; *n* = 50 2. Sertraline (50–100 mg/day); *n* = 50	-90 -10	L-DLPFC	-60 -4 -15	-2400 -10 -24000	4
Fu et al., 2022 (China)	104	-ICD-10 -Inpatients	15.5 (12–18)	-5.7 -27.9	4	1. SSRIs^d^ + rTMS; *n* = 52 2. SSRIs^d^ + sham; *n* = 52	-100 -1	R-DLPFC	-40 -5 -20	-2000 -20 -40000	5
Lu et al., 2020 (China)	116	-ICD-10 -Inpatients	14.2 (12–18)	-5.3 -47.2	4	1. Sertraline (100–150 mg/day) + rTMS; *n* = 58 2. Sertraline (100–150 mg/day) + sham; *n* = 58	-80 -10	L-DLPFC	-30 -2 -28	-NR -20 -NR	3
Ma et al., 2021 (China)	80	-ICD-10 -NR	16.1 (13–18)	-NR -51.3	6	1. Sertraline (50 mg/day) + rTMS; *n* = 40 2. Sertraline (50 mg/day); *n* = 40	-80 -10	L-DLPFC	-NR -1 -20	-NR -30 -NR	3
Zhang et al., 2019 (China)	40	-DSM-IV -Inpatients	15.7 (13–17)	-10.8 -36.4	6	1. Fluoxetine (20 mg/day) + rTMS; *n* = 20 2. Fluoxetine (20 mg/day) + sham; *n* = 20	-100 -1	R-DLPFC	NR	-1200 -30 -36000	5
Zhu et al., 2021 (China)	122	-CCMD-3 -Inpatients	14.5 (12–18)	-5.6 -37.7	4	1. Sertraline (100–150 mg/day) + rTMS; *n* = 61 2. Sertraline (100–150 mg/day); *n* = 61	-80 -10	L-DLPFC	-30 -2 -28	-NR -20 -NR	2

a Data were extracted based on random assignment.

b Available data were extracted based on the mean baseline value of each included trial.

c The treatment duration was defined as the entire period from begin of the first rTMS treatment to the endpoint of the last rTMS treatment.

d Participants of active or sham groups were treated with selective serotonin reuptake inhibitors, but the authors did not specify which antidepressant they received.

CCMD-3, Chinese Classification and Diagnostic Criteria of Mental Disorders 3rd edition; DSM-IV, Diagnostic and Statistical Manual of Mental Disorders 4th edition; ICD-10, International Statistical Classification of Diseases and Related Health Problems 10th revision; L-DLPFC, left dorsolateral prefrontal cortex; MT, motor threshold; NR, not reported; R-DLPFC, right dorsolateral prefrontal cortex; rTMS, repetitive transcranial magnetic stimulation; s, second; SSRIs, selective serotonin reuptake inhibitors; yrs, years.

### 3.3. Quality assessment

As displayed in Supplementary Figure 1, four RCTs (4/6, 66.7%) were rated as ‘low risk’ regarding random sequence generation. Three RCTs (3/6, 50.0%) were rated ‘low risk’ regarding the blinding of participants and personnel the blinding of outcome assessment. Selective reporting was rated as ‘low risk’ in all of the included RCTs. The mean Jadad score was 3.7 (range = 2–5), and five out of the six RCTs (5/6, 83.3%) were classified as high-quality studies (Jadad score ≥ 3) (Table 1). Following the GRADE approach (Supplementary Table 2), the overall evidence quality was rated as ‘low’ (2/8, 25%), ‘moderate’ (5/8, 62.5%) and ‘high’ (1/8, 12.5%).

### 3.4. Primary outcomes

**Figure 2:**
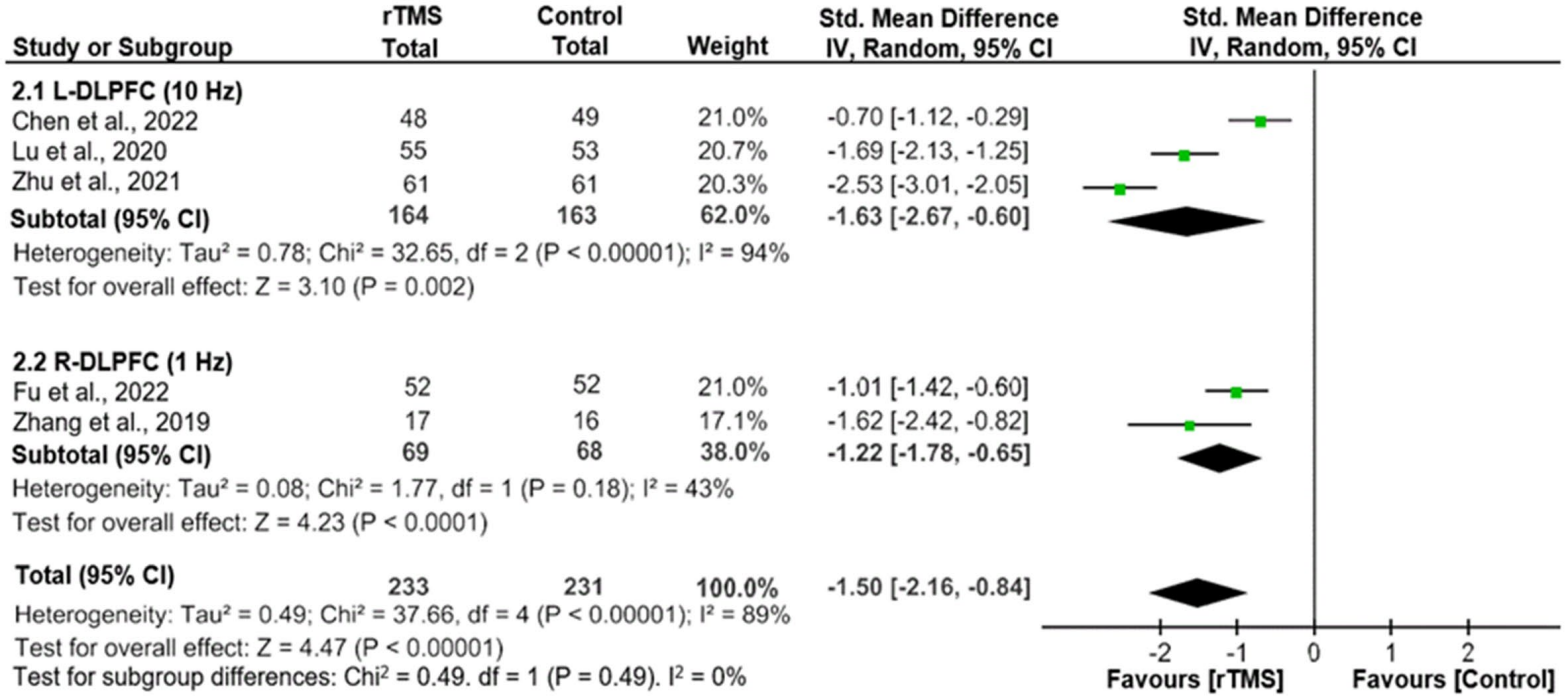
Adjunctive rTMS for adolescents with FE-MDD: forest plot for the improvement of depressive symptoms assessed by the HAMD. CI, confidence interval; FE-MDD, first-episode major depressive disorder; HAMD, Hamilton Depression Scale; L-DLPFC, left dorsolateral prefrontal cortex; R-DLPFC, right dorsolateral prefrontal cortex; rTMS, repetitive transcranial magnetic stimulation.

As shown in Figure 2, adjunctive active rTMS outperformed the control group in improving depressive symptoms (5 RCTs, *n* = 464, SMD = -1.50, 95% CI: −2.16, −0.84; *I*^2^ = 89%, *p* < 0.00001), as measured by the HAMD-24 (3 RCTs) (20, 21, 23) and HAMD-17 (2 RCTs) (19, 24). Similarly, significant findings remained in a sensitivity analysis after excluding one RCT with an outlying effect size (23) (4 RCTs, *n* = 342, SMD = −1.22, 95% CI: −1.70, −0.73; *I*^2^ = 75%, *p* < 0.00001). In addition, the superiority of adjunctive rTMS was retained when divided into two subgroups by frequency, which included HF-rTMS (3 RCTs, *n* = 327, SMD = −1.63, 95% CI: −2.67, −0.60; *I*^2^ = 94%, *p* = 0.002) and LF-rTMS (2 RCTs, *n* = 137, SMD = -1.22, 95% CI: −1.78, −0.65; *I*^2^ = 43%, *p* < 0.0001).

### 3.5. Secondary outcomes

#### 3.5.1. Study-defined response and remission

Adjunctive rTMS was superior to the control group regarding response (4 RCTs, *n* = 406; RR: 1.35, 95% CI: 1.04, 1.76; *I*^2^ = 56%, *p* = 0.03) and remission (3 RCTs, *n* = 306; RR: 1.35, 95% CI: 1.03, 1.77; *I*^2^ = 0%, *p* = 0.03) (Table 2).

**Table 2:** Adjunctive rTMS for adolescents with FE-MDD: secondary outcomes.

Variables	Number of studies (sample size)	RRs/SMDs	95% CI [Lower, Upper]	*I^2^* (%)	*P*
Study-defined response and remission					
Response	4 (406)	1.35	[1.04, 1.76]	56	**0.03**
Remission	3 (306)	1.35	[1.03, 1.77]	0	**0.03**
Discontinuation rate					
Discontinuation due to any reasons	6 (562)	0.79	[0.32, 1.93]	0	0.60
Adverse events					
Headache	4 (324)	3.07	[0.70, 13.37]	0	0.14
Loss of appetite	2 (180)	0.43	[0.07, 2.88]	0	0.39
Dizziness	2 (184)	1.30	[0.14, 12.29]	29	0.82
Nausea	2 (120)	0.26	[0.03, 2.24]	0	0.22

*P* < 0.05 is in bold. CI, confidence interval; FE-MDD, first-episode major depressive disorder; RRs, risk ratios; rTMS, repetitive transcranial magnetic stimulation; SMDs, standardized mean differences.

#### 3.5.2. Discontinuation due to any reason

As shown in Table 2, discontinuation due to any reason was similar between the two groups (6 RCTs, *n* = 562, RR = 0.79, 95% CI: 0.32, 1.93; *I*^2^ = 0%, *p* = 0.60). The reasons for discontinuation of each included RCT were summarized in Supplementary Table 2.

#### 3.5.3. Adverse events

Four studies (19, 20, 22, 24) reported adverse events. As displayed in Table 2, no significant differences were found regarding adverse events, including headache, loss of appetite, dizziness and nausea (*p* = 0.14–0.82).

#### 3.5.4. Neurocognitive function

Five out of six RCTs (83.3%, 5/6) (19–21, 23, 24) examine the effect of adjunctive rTMS on neurocognitive function in adolescents with FE-MDD. Among them, 4 RCTs (80.0%, 4/5) (19–21, 23) found that active rTMS group outperformed the comparator in improving neurocognitive function as measured by different measurement tools (Table 3). However, one RCT (20.0%, 1/5) (24) found no significant differences between the two groups.

**Table 3:** Adjunctive rTMS for adolescents with FE-MDD: neurocognitive function.

Study	Neurocognitive function	Findings
Chen et al., 2022	IVA-CPT THINC-it	Compared with sham stimulation, rTMS can significantly improve neurocognitive function including the attention quotient (listening, visual and full-scale) of IVA-CPT as well as the Spotter of THINC-it in adolescents with FE-MDD (all *p* < 0.05).
Fu et al., 2022	MoCA TMT-A	Compared with sham stimulation, rTMS can significantly improve neurocognitive function as measured by the MoCA and TMT-A in adolescents with FE-MDD (all *p* < 0.05).
Lu et al., 2020	WCST TMT	Compared with sham stimulation, rTMS can significantly improve neurocognitive function as measured by the WCST and TMT in adolescents with FE-MDD (all *p* < 0.05).
Ma et al., 2021	NR	NR
Zhang et al., 2019	CPT SCWT MCCB	No differences were found regarding the CPT, SCWT and MCCB tests between active rTMS and sham stimulation group in adolescents with FE-MDD (all *p* > 0.05).
Zhu et al., 2021	MoCA CMS^a^	Compared with the control group, adjunctive rTMS appeared to be effective in improving neurocognitive functions measured with MoCA and CMS in adolescents with FE-MDD (*p* < 0.05).

a An assessment scale for memory function adapted for the Chinese population compiled and revised by the Institute of psychology, Chinese Academy of Sciences.

CMS, Clinical Memory Scale; CPT, continuous performance test; IVA-CPT, Integrated Visual and Auditory Continuous Performance Test; MCCB, MATRICS Consensus Cognitive Battery; MoCA, Montreal Cognitive Assessment; MT, motor threshold; rTMS, repetitive transcranial magnetic stimulation; SCWT, Stroop Word-Color Interference Test; TMT, Trail Making Test; TMT-A, Trail Making Test-A; WSCT, Wisconsin Card Sorting Test.

### 3.6. Publication bias

Given that the number of included RCTs was less than 10, publication bias could not be analyzed as recommended (39).

## 4. Discussion

To the best of our knowledge, this is the first meta-analysis to examine the efficacy, tolerability and safety of rTMS as an adjunct treatment for adolescents (12–18 years) with FE-MDD. Six RCTs (19–24) involving 562 adolescents with FE-MDD were included in this meta-analysis. The main findings are as follows: (1) adjunctive rTMS was superior in improving depressive symptoms over the control group; (2) adolescents with FE-MDD treated with rTMS had a significantly greater response and remission over the control group, suggesting that rTMS may have beneficial effects for adolescents with FE-MDD; (3) rTMS appeared to be safe and tolerable as an adjunct treatment for adolescents with FE-MDD; and (4) adjunctive rTMS appears to be effective in improving neurocognitive function in adolescents with FE-MDD.

Although no meta-analysis has investigated the therapeutic effects, tolerability and safety of adjunctive rTMS for adolescents with FE-MDD, several systematic reviews (31, 40, 41) have preliminarily explored the efficacy of adjunctive rTMS for adolescents with MDD. For example, a systematic review (40) found that rTMS could reduce depressive symptoms in adolescents with MDD. However, this systematic review (40) included RCTs consisting of a mixture of FE-MDD and multiepisode MDD. Additionally, a systematic review (41) also suggested that rTMS is an effective and well-tolerated treatment for adolescents with TRD. Therefore, the findings of our study provided further support for the utility of rTMS treatment (either HF-rTMS or LF-rTMS) combined with antidepressants for adolescents with FE-MDD. Importantly, adjunctive rTMS appeared to be safe and tolerable for adolescents or adults with FE-MDD (16).

The underlying mechanism of the effect of rTMS on depressive symptoms may be that it can generate repeated pulses that act on the cerebral cortex and then transform neural functional activities in the brain circuits related to the pathophysiology of depression (42, 43). More specifically, HF-rTMS (> 5 Hz) has an excitatory effect on neural functional activities, and LF-rTMS (≤ 1 Hz) has the opposite effect on depression, which is characterized by reduced neural functional activity in the L-DLPFC and increased neural functional activity in the R-DLPFC (40). Previous research has found that HF-rTMS on L-DLPFC and LF-rTMS on R-DLPFC both have similar mechanisms that induce equivalent functional changes in the brain associated with antidepressant efficiency in MDD patients, including a decrease in brain limbic activity within the left perirhinal cortex (44). In addition, rTMS has a certain potential for modulating pathologic imbalances in GABAergic and glutamatergic neurocircuitry (45, 46), which play an important role in depression (47, 48).

Previous meta-analyses have found that rTMS appears to be effective in improving neurocognitive function in adults with FE-MDD (49, 50). For example, Martin et al. (49) found that rTMS courses administered to the prefrontal cortex for depression may produce modest neurocognitive enhancing effects specific to psychomotor speed, visual scanning, and set-shifting ability. A possible reason is that the improvement of neurocognitive function may be a secondary effect after emotional improvement (49). Although the findings of the neurocognitive effects of adjunctive rTMS for adolescents with FE-MDD are mixed in the included five RCTs (19–21, 23, 24), four out of five RCTs (80.0%) found the significant superiority of adjunctive rTMS over the comparator in improving neurocognitive function after rTMS (19–21, 23). Only one RCT (24) included in this meta-analysis found no deterioration or significant improvement in neurocognitive function with a small sample size (*n* = 40). Taken together, adjunctive rTMS appears to be effective in improving neurocognitive function in adolescents with FE-MDD, although further studies focusing on adjunctive rTMS on neurocognitive function in adolescents with FE-MDD are warranted.

There are several limitations of this present study. First, the sample size of the meta-analysis was relatively small (*n* = 562), which might reduce the statistical power. Second, all of the included RCTs had relatively short observation periods (2–6 weeks) and lacked long-term follow-up. Third, all included studies were conducted in China and involved only Chinese adolescents. Thus, the findings of the present study are not generalizable to other countries or populations. Fourth, the confounding effects of antidepressant medications could not be detected due to insufficient information in the included studies. Fifth, the significant heterogeneity for primary outcome (*I*^2^ = 89%) remained, even in a sensitivity analysis (*I*^2^ = 75%), which may partly attribute to the significant heterogeneity of the rTMS protocols used in the included RCTs.

## 5. Conclusion

Adjunctive rTMS appears to be a beneficial strategy in improving depressive symptoms and neurocognitive function in adolescents with FE-MDD. Higher quality RCTs with larger sample sizes and longer follow-ups are warranted in the future.

## Data availability statement

The original contributions presented in the study are included in the article/Supplementary material, further inquiries can be directed to the corresponding authors.

## Author contributions

C-HS, Z-MS, J-XM, and X-HY selected studies and extracted the data. WZ from (Guangzhou) reviewed all the data and helped to mediate disagreements. C-HS wrote the first draft. All authors contributed to the article and approved the submitted version.

## Funding

This study was funded by the National Natural Science Foundation of China (82101609), Scientific Research Project of Guangzhou Bureau of Education (202032762), Guangzhou Health Science and Technology Project (20211A011045), Guangzhou Science and Technology Project of traditional Chinese Medicine and integrated traditional Chinese and Western medicine (20212A011018), China International Medical Exchange Foundation (Z-2018-35-2002), Science and Technology Program Project of Guangzhou (202102020658), the Science and Technology Program of Guangzhou (2023A03J0839 and 2023A03J0436), Science and Technology Planning Project of Liwan District of Guangzhou (202201012), The Natural Science Foundation Program of Guangdong (2023A1515011383), Guangzhou Municipal Key Discipline in Medicine (2021−2023), Guangzhou High-level Clinical Key Specialty, and Guangzhou Research-oriented Hospital. The funders had no role in study design, data collection and analysis, decision to publish, or preparation of the manuscript.

## Conflict of interest

The authors declare that the research was conducted in the absence of any commercial or financial relationships that could be construed as a potential conflict of interest.

## Publisher’s note

All claims expressed in this article are solely those of the authors and do not necessarily represent those of their affiliated organizations, or those of the publisher, the editors and the reviewers. Any product that may be evaluated in this article, or claim that may be made by its manufacturer, is not guaranteed or endorsed by the publisher.

## Appendix S1. Methods

(“Transcranial Magnetic Stimulation”[MeSH] OR Transcranial Magnetic Stimulation OR rtms OR tms) AND (“depression”[MeSH] OR depression OR depressive OR depressed OR melancholia) AND (child OR childhood OR children OR adolescent OR adolescents OR puberty OR pubertal OR juvenile OR teen* OR youth OR preschool OR preschool child OR school age OR high school OR student OR pediatric* OR paediatric* OR minors OR boys OR boy OR girl*) AND (first episode OR early phase OR early-phase OR FEP OR recent onset OR untreated OR unmedicated OR non medicated OR undiagnosed OR first diagnosed OR first diagnosis OR drug-free OR antidepressant-free OR medication-free OR drug-naïve OR antidepressant-naïve OR medication-naïve OR treatment-naïve OR never-medicated) AND (random OR random* OR control OR RCT).
