# Supplementary material for: Adjunctive repetitive transcranial magnetic stimulation for adolescents with first-episode major depressive disorder: a meta-analysis
Source: Front Psychiatry. 2023 Aug 1;14:1200738. doi: 10.3389/fpsyt.2023.1200738 (PMC10428010; doi:10.3389/fpsyt.2023.1200738)
Supplement: Supplementary file 1 [file Data_Sheet_1.docx]

**Supplemental Table 1. GRADE analyses: adjunctive rTMS for adolescents with FE-MDD.**

| **Primary** and *secondary outcomes* | Number of study (subjects) | Risk of bias | Inconsistency | Indirectness | Imprecision | Publication bias | Large effect | Overall quality of evidence^a^ |
| --- | --- | --- | --- | --- | --- | --- | --- | --- |
| **The improvement of depressive symptoms** | 5 (464) | Serious^b^ | Serious^c^ | No | No | Undetected | No | +/+/-/-; Low |
| *Response rate* | 4 (406) | Serious^b^ | Serious^c^ | No | No | Undetected | No | +/+/-/-; Low |
| *Remission rate* | 3 (306) | Serious^b^ | No | No | No | Undetected | No | +/+/+/-; Moderate |
| *Discontinuation due to any reasons* | 6 (562) | Serious^b^ | No | No | No | Undetected | No | +/+/+/-; Moderate |
| *Headache* | 4 (324) | No | No | No | No | Undetected | No | +/+/+/+; High |
| *Loss of appetite* | 2 (180) | No | No | No | Serious^d^ | Undetected | No | +/+/+/-; Moderate |
| *Dizziness* | 2 (184) | No | No | No | Serious^d^ | Undetected | No | +/+/+/-; Moderate |
| *Nausea* | 2 (120) | No | No | No | Serious^d^ | Undetected | No | +/+/+/-; Moderate |
| ^a^GRADE Working Group grades of evidence: High quality=further research is very unlikely to change our confidence in the estimate of effect. Moderate quality=further research is likely to have an important impact on our confidence in the estimate of effect and may change the estimate. Low quality=further research is very likely to have an important impact on our confidence in the estimate of effect and is likely to change the estimate. Very low quality=we are very uncertain about the estimate. ^b^The trial blinding used for one study was wrong  ^c^ Meta-analytic results presented a serious inconsistency when I^2^ values were greater than 50% or *p*<0.1 in the Q statistics.  ^d^ For dichotomous outcomes, N<300.  Abbreviations: GRADE=Grading of Recommendations Assessment, Development, and Evaluation; FE-MDD=first-episode major depressive disorder; rTMS=repetitive transcranial magnetic stimulation. | | | | | | | | |

**Supplemental Table 2. The reasons for discontinuation of rTMS group and control group**

|  | **Discontinuation due to any reasons**  **(n)** | **Headache**  **(n, %)** | **Side effects of medication**  **(n, %)** | **Refusal from family**  **(n, %)** | **Incomplete assessment**  **(n, %)** | **Hypomanic episode**  **(n, %)** | **Not reported**  **(n, %)** |
| --- | --- | --- | --- | --- | --- | --- | --- |
| rTMS group | 8 | 3 (37.5) | 0 | 0 | 2 (25.0) | 0 | 3 (37.5) |
| Control group | 10 | 0 | 2 (20.0) | 1 (10.0) | 1 (10.0) | 1 (10.0) | 5 (50.0) |
| Abbreviations: rTMS=repetitive transcranial magnetic stimulation | | | | | | | |

**Supplemental Figure 1: Risk of bias.**

|  | ***Random sequence generation (selection bias)*** | ***Allocation concealment (selection bias)*** | ***Blinding of participants and personnel*** | ***Blinding of outcome assessment (Symptom reduction, response)*** | ***Incomplete outcome data addressed (attrition bias)*** | ***Selective reporting (reporting bias)*** | ***Other sources of bias*** |
| --- | --- | --- | --- | --- | --- | --- | --- |
| **Chen et al., 2022** | + | ？ | ？ | ？ | + | + | ? |
| **Fu et al., 2022** | + | ? | + | + | + | + | ? |
| **Lu et al., 2020** | - | ? | + | + | ？ | + | ? |
| **Ma et al., 2021** | + | ？ | ？ | ？ | + | + | ? |
| **Zhang et al., 2019** | + | ? | + | + | + | + | ? |
| **Zhu et al., 2021** | ？ | ? | - | ? | + | + | ? |

+: Low risk of bias, -: High risk of bias, ?: Unclear risk of bias
